# Uncovering Sex and Gender Differences in Sarcoidosis: A Systematic Review of Current Evidence

**DOI:** 10.3390/jpm16010024

**Published:** 2026-01-05

**Authors:** Tommaso Pianigiani, Beatrice Perea, Akter Dilroba, Asia Fanella, Clarissa Milli, Sara Postiferi, Leonardo Rubegni, Laura Bergantini, Miriana D’Alessandro, Paolo Cameli, Elena Bargagli

**Affiliations:** Respiratory Diseases Unit, Department of Medical, Surgical and Neurosciences, University of Siena, 53100 Siena, Italy

**Keywords:** sarcoidosis, gender differences, personalised medicine

## Abstract

**Introduction:** Sarcoidosis is a systemic granulomatous disorder classified among interstitial lung diseases (ILDs). While the lungs and intrathoracic lymph nodes are most affected, the disease can involve multiple organs. The heterogeneity of clinical presentation arises from complex interactions between environmental exposures and immune responses in genetically susceptible individuals. Sex-dependent genetic variations are associated with differences in phenotype and organ localization. Gender-related factors also influence the impact of sarcoidosis on quality of life and health perception, contributing to variability in disease burden and outcomes. **Aim of the study:** to provide an overview of sex- and gender-related differences in sarcoidosis, focusing on pathophysiological and clinical implications. **Material and Methods:** The systematic search was conducted on Medline database through Pubmed search engine. We included all clinical studies from 1992 to the present, and imposed language restrictions, accepting only English publications. Case reports, reviews, and pre-print studies were excluded. **Results:** A total of 35 studies were included. Sex differences significantly influenced both age of onset and clinical presentation of the disease. Women received a diagnosis of sarcoidosis at an older age and exhibited more frequently extrapulmonary localizations, with predominant involvement of the eyes, skin, and extra-thoracic lymph nodes. In contrast, men more commonly presented with limited pulmonary forms. Löfgren syndrome was more prevalent among women and appeared to be associated with sex-specific genetic variations, particularly within the MHC region. Gender differences also impacted quality of life and disease perception: women reported a lower quality of life and were more susceptible to anxiety and depression throughout the disease course. **Conclusions:** This report confirms that clinical presentation of sarcoidosis is significantly influenced by sex and gender. The identification of sex- and gender-specific clinical patterns supports a personalized medicine framework, in which diagnostic assessment, monitoring strategies, and therapeutic approaches may be tailored according to individual biological and gender-related characteristics.

## 1. Introduction

Sarcoidosis is a systemic inflammatory disease of unknown aetiology, characterized by the formation of non-caseating granulomas in multiple organs [1,2]. Incidence, clinical presentation, organ involvement, and disease course vary significantly according to ethnicity, biological sex, and age [3,4]. Intrathoracic involvement, seen in nearly 90% of cases, is the most common presentation and often leads to incidental diagnosis due to bilateral hilar lymphadenopathy and parenchymal micronodules along lymphatic pathways [5,6].

Extrapulmonary manifestations of sarcoidosis are diverse and can involve the skin, eyes, liver, spleen, kidneys, and potentially any organ [7,8,9]. Among skin lesions, purple-red or brown papules are relatively common, whereas lupus pernio, although distinctive, is rare. Ocular involvement includes uveitis and xerophthalmia, and renal manifestations may present as nephrolithiasis due to calcium metabolism abnormalities. Extra thoracic lymphadenopathy is also observed, reflecting the systemic nature of the disease [10,11,12].

Over half of patients experience a self-limiting disease with spontaneous remission, whereas about 25% develop chronic or relapsing disease requiring long-term therapy [13]. A smaller proportion may present with severe pulmonary, cardiac, or neurologic involvement, associated with increased mortality risk [14,15].

Diagnosis remains one of exclusion and is established based on three essential criteria: (1) clinical and/or radiologic findings consistent with sarcoidosis; (2) histologic evidence of non-caseating granulomas; and (3) exclusion of other potential causes [16]. In specific cases, such as Lofgren’s syndrome (fever, bilateral hilar lymphadenopathy, erythema nodosum, and/or polyarthralgia), diagnosis may be established without the need for a biopsy [4,17,18].

Positron emission tomography (PET) can be useful for assessing the extent and metabolic activity of sarcoidosis, particularly in cases with suspected cardiac involvement or when identifying sites of active disease for biopsy. However, PET is not routinely used in the management of most patients with sarcoidosis due to its limited availability, high cost, potential for nonspecific uptake, and the fact that conventional imaging and clinical evaluation are sufficient for the majority of cases [19,20,21].

Sarcoidosis typically affects young adults, particularly women aged 20–39 years [22,23], with higher prevalence in Scandinavian countries [22,24,25] and greater disease burden among Black individuals in the United States, who more often experience extrapulmonary and chronic-relapsing clinical course of disease [6,24,26,27].

These epidemiologic and clinical differences have raised important questions about the pathophysiology of sarcoidosis and the influence of biological sex and gender-related factors on disease incidence and expression [23].

Gender medicine explores how biological sex and sociocultural gender influence disease prevention, diagnosis, treatment, prognosis, and psychosocial impact. Integrating sex- and gender-based perspectives strengthens scientific evidence and fosters appropriateness and equity in healthcare [28,29,30].

In this context, biological sex refers to physiological differences between males and females—such as chromosomal patterns, hormone levels, and anatomical features—while gender encompasses socially constructed roles, identities, behaviours, and relationships, shaped by cultural and socioeconomic factors [31,32].

Building on these definitions, the comprehension of sex- and gender-related disparities in sarcoidosis is pivotal for advancing personalised and precision medicine. Stratifying patients according to biological sex, gender, genetic background, and hormonal status may support the development of individualized diagnostic, monitoring, and therapeutic pathways, ultimately improving prognostic accuracy.

In this context, the aim of this systematic review is to analyse the available literature on sex- and gender-associated differences in sarcoidosis, elucidating how biological and sociocultural factors influence disease onset, clinical presentation, and patient-reported outcomes. By synthesizing current evidence, this review highlights the relevance of integrating both sex and gender as key modifiers of disease heterogeneity and underscores their potential to inform more tailored and equitable approaches to sarcoidosis management.

## 2. Methods

### 2.1. Data Collection and Analysis

This systematic review was conducted according to the Preferred Reporting Items for Systematic Reviews and Meta-analyses (PRISMA) guidelines (the PRISMA checklist see Supplementary Material File S1) [33].

#### 2.1.1. Search Strategy

The literature search was performed in the MEDLINE database via PubMed, Embase, Scopus, and Web of Science. The final search was conducted on 8 December 2025.

The following search terms were used, combined with Boolean operators: (“sarcoidosis” AND (“sex” OR “gender”)). The search was restricted to studies published in the English language and conducted in humans. Studies published from 1990 to 8 December 2025 were considered eligible. Case reports, editorials, narrative reviews, systematic reviews, and pre-print articles were excluded.

#### 2.1.2. Eligibility Criteria

Inclusion criteria:

- Original clinical studies (randomized controlled trials, prospective or retrospective observational studies)
- Multicentric or monocentric study design
- Enrolment of patients with an established diagnosis of sarcoidosis
- Full-text availability
- Reporting data on biological sex, gender, and/or gender-related clinical outcomes
- No age restrictions were applied

Exclusion criteria:

- Case reports
- Editorials
- Narrative reviews or systematic reviews
- Pre-print articles
- Studies not reporting outcomes relevant to sex- or gender-related differences

#### 2.1.3. Study Selection Process

The study selection was performed by two independent reviewers. Titles and abstracts were first screened to identify potentially eligible studies. Subsequently, the full texts of all selected articles were independently assessed for eligibility according to the predefined inclusion and exclusion criteria. Disagreements between reviewers were resolved by consensus.

#### 2.1.4. Data Extraction

Data were extracted independently by two reviewers using a standardized data extraction form.

The following variables were collected:

- Study design
- Sample size
- Age and sex distribution
- Clinical presentation
- Quality of life measures
- Illness perception outcomes

#### 2.1.5. Risk of Bias Assessment

After full-text screening, all eligible studies were assessed for risk of bias using the Newcastle–Ottawa Scale (NOS) for observational studies. No minimum sample size based on formal power calculation was applied; studies were excluded only if they were classified as at high risk of bias according to the NOS (≤3 stars). The scale evaluates three domains: Selection (maximum 4 stars), Comparability (maximum 2 stars), and Outcome (maximum 3 stars), with a total possible score of 9 stars. Studies scoring 7–9 stars were considered at low risk of bias, 4–6 stars at moderate risk, and ≤3 stars at high risk of bias. Studies scoring ≤3 stars were excluded from the analysis. The assessment was performed independently by two reviewers, and disagreements were resolved by consensus. A detailed summary of the risk-of-bias assessment is provided in the Supplementary Material Table S1.

#### 2.1.6. Data Synthesis

The included studies were divided into two groups based on their main outcomes:

- Biological sex and disease presentation
- Gender differences in illness perception and quality of life

**Figure 1:**
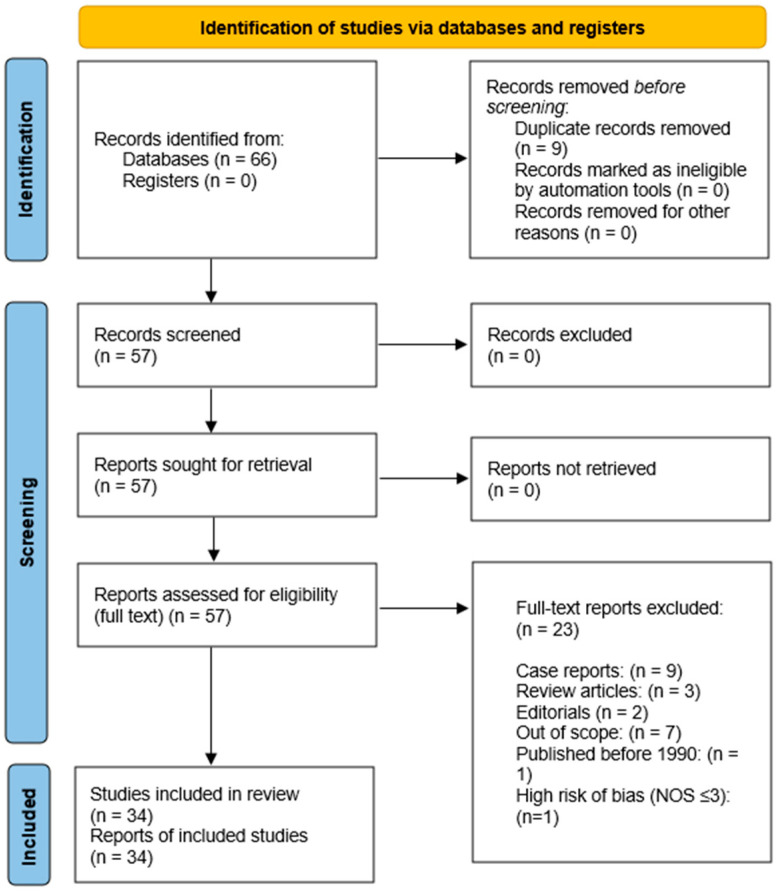
PRISMA 2020 flow diagram of study selection. A total of 66 records were identified through database searching. After removal of 9 duplicates, 57 records were screened and assessed for full-text eligibility. Twenty-three articles were excluded for the following reasons: case reports (n = 9), review articles (n = 3), editorials (n = 2), studies out of scope (n = 7), studies published before 1990 (n = 1), and high risk of bias according to the Newcastle–Ottawa Scale (n = 1). Ultimately, 34 studies were included in the qualitative synthesis.

Figure 1 shows the flow diagram depicting study selection and final inclusion in the review.

### 2.2. Protocol Registration

This systematic review was not prospectively registered in PROSPERO because the protocol was developed internally prior to the initiation of the literature search. However, the methodology was strictly predefined and adhered to throughout the study.

## 3. Results

A total of 57 studies were selected in the first phase of article collection. Thirty-four studies (22 observational retrospective studies, 7 observational prospective studies and 5 cross sectional trials) were selected for final analysis, as depicted in Figure 1 and listed in Table 1. We excluded 23 studies because the study design was unsuitable for this study’s purpose (see Table S2).

### 3.1. Biological Sex and Disease Presentation

#### 3.1.1. Sex Differences in Age at Disease Onset

Biological sex is a significant determinant of both the age at disease onset and the clinical manifestations of sarcoidosis. Based on multiple studies analysing different patient cohorts, women tend to have an older age at diagnosis and at disease onset [34,35,36,37]. In some cases, a bimodal distribution of disease onset has also been observed in female patients [38,39].

In a population-based cohort from Olmsted County, US, the age at diagnosis was significantly higher among women than men (48.3 vs. 42.8 years; *p* < 0.001), despite identical sex distribution in incidence, confirming that females develop clinically recognized sarcoidosis at an older age than males [36]. Similarly, in a large Swedish register of 1429 patients, median age at diagnosis was 43 years in women versus 40 years in men (*p* < 0.01), and a clear bimodal age distribution was observed in women—with a second peak around 50–60 years—that was not seen in men [34]. Data from an Estonian cohort further support this pattern, showing that female patients were on average 8.5 years older than males at first referral for sarcoidosis, and that older age remained independently associated with female sex after multivariable adjustment [35]. Finally, in a French comparative study specifically addressing late-onset sarcoidosis (diagnosis ≥ 65 years), the mean age at diagnosis was 70.6 years and 83% of late-onset cases occurred in women, underscoring a marked female predominance in very late disease onset [38]. Further support for a female-predominant late-onset pattern derives from a large prospective U.S. cohort of 116,430 women followed for 22 years (Nurses’ Health Study II), in which the median age at diagnosis among incident cases was 48 years (range 28–63 years), and age-specific incidence rates increased significantly with advancing age, reaching the highest values in women aged 55 years or older [39].

#### 3.1.2. Sex Differences in Extrapulmonary Organ Involvement

Extrapulmonary involvement of sarcoidosis is generally more common in women [34,35,36]. Notably, sex-related differences have been reported also in the incidence and expression of organ-specific extrapulmonary manifestations.

##### Skin Involvement

Cutaneous manifestations, including erythema nodosum and lupus pernio, are also more frequently observed in women [40,41].

This female predominance of cutaneous involvement is consistently confirmed in large dermatological series. In a Turkish cohort of 170 sarcoidosis patients with skin involvement, women accounted for 80% of cases, a proportion significantly higher than in sarcoidosis patients without cutaneous manifestations (*p* < 0.001), with erythema nodosum representing the most frequent lesion, followed by plaques, subcutaneous nodules, scar lesions and lupus pernio [40].

Similarly, in a Taiwanese hospital-based series of biopsy-proven cutaneous sarcoidosis, a striking female predominance was observed (female-to-male ratio 4.4:1), with women being significantly older at diagnosis than men (55.6 vs. 41.7 years; *p* = 0.037), further supporting a strong sex-related susceptibility to skin involvement [41].

Sex-specific patterns are also evident in acute sarcoidosis phenotypes: in Löfgren’s syndrome, erythema nodosum was found predominantly in women (67%), whereas men more frequently presented with isolated periarticular ankle inflammation without erythema nodosum (*p* < 0.0001), despite otherwise similar clinical and radiological features [42].

##### Ocular Involvement

Ocular involvement, particularly uveitis, has been shown to be more prevalent in women as well [43,44,45,46,47]. Large ophthalmologic series consistently confirm that sarcoidosis-related uveitis shows a clear female predominance across different geographic areas. In the Japanese cohort of 1240 patients with intraocular inflammation, sarcoidosis was the most frequent etiology and women accounted for 72.4% of sarcoid uveitis cases, with a significantly higher mean age at ocular disease onset compared with men (50.3 ± 16.5 vs. 35.1 ± 19.0 years; *p* < 0.0001), and a bimodal age distribution with a second peak between 50 and 70 years observed only in women [44]. Similarly, in a cohort of 60 Chinese patients with biopsy-proven sarcoidosis presenting with uveitis, a marked female predominance was observed (male-to-female ratio 1:6.5), with a peak incidence of ocular onset in the sixth decade of life, confirming that ocular sarcoidosis in Asian populations predominantly affects middle-aged to older women [45]. In this same series, uveitis represented the initial manifestation of sarcoidosis in 68% of patients and was bilateral in 90%, underscoring the high diagnostic relevance of ocular involvement—particularly in women—for the identification of systemic disease [45]. These findings are in line with other ophthalmologic studies reporting frequent bilateral involvement, a predominance of anterior uveitis and panuveitis, and a substantial burden of posterior segment complications, which largely determine visual prognosis [43,44,45,46,47].

##### Cardiac Involvement

Sex-related differences in cardiac sarcoidosis have been documented in terms of both incidence and clinical presentation. While some studies suggest a higher frequency of cardiac involvement in men [48,49], findings remain inconsistent across the literature. This heterogeneity likely reflects differences in study design, diagnostic criteria, and the ethnicity of the populations examined. In a cohort study by Kalra et al., women reported more frequent symptoms such as chest pain and palpitations compared to men. However, cardiac magnetic resonance imaging revealed less extensive cardiac involvement in women, as well as a lower prevalence of dyspnoea, syncope, presyncope, and arrhythmias at presentation. Despite these differences, overall mortality did not differ between sexes, although women experienced a significantly lower burden of ventricular arrhythmias [50]. Consistent with these findings, a multicentric study by Iso et al. reported a higher risk of potentially fatal ventricular arrhythmic events in men, who also showed reduced left ventricular ejection fraction (LVEF) and a greater risk of comorbidities, including hypertension, atrial fibrillation, and coronary artery disease [51]. Conversely, Duvall et al. reported more nuanced findings in a population stratified by biological sex and race. While white male patients exhibited a higher incidence of ventricular arrhythmias and a more frequent need for implantable cardioverter-defibrillator (ICD) implantation, no significant differences in baseline LVEF were observed between sexes, suggesting that factors beyond systolic dysfunction, at least if evaluated through transthoracic echocardiography, may underlie the higher arrhythmic risk in men [52]. The outcome of ICD implantation was also addressed by Ahmed et al., whose retrospective study confirmed a greater ICD implantation rate and a higher risk of sudden cardiac death in the male population [53]. Finally, the multicentre study by Nakasuka et al. focused on patients undergoing cardiac resynchronization therapy and reported a lower incidence of adverse cardiac events and ventricular arrhythmias in women, suggesting a potentially more favourable cardiac prognosis in the female population [54].

##### Other Organ Involvement

Beyond cutaneous, ocular and cardiac involvement, sex-related differences have also been reported for other extrapulmonary localizations, including extra thoracic lymph nodes, liver, spleen and the musculoskeletal system. Extra thoracic lymphadenopathy represents one of the most frequent systemic sites of disease dissemination and appears to be more commonly observed in women across different clinical cohorts, often in association with a more disseminated extrapulmonary phenotype [42,55]. Hepatic and splenic involvement are generally less symptomatic and frequently detected incidentally during systemic staging; available cohort studies do not consistently demonstrate robust sex-related differences for these sites, although they tend to cluster within more multisystemic disease forms, which are overall more common in women [35,55].

Sex-specific patterns are also evident in acute sarcoidosis phenotypes. In Löfgren’s syndrome, erythema nodosum and bilateral ankle arthritis—often associated with hilar lymphadenopathy—are significantly more frequent in women, whereas men more commonly present with isolated periarticular ankle inflammation without erythema nodosum, despite similar radiographic staging [42]. Salivary gland involvement, although relatively uncommon, has likewise been reported more frequently in female patients and typically occurs in the context of chronic multisystem disease [55,56].

Musculoskeletal involvement—including both osseous and skeletal muscle sarcoidosis—does not represent a rare manifestation per se, but remains an uncommon and often underdiagnosed localization in most clinical series [56,57]. When present, it typically manifests as either nodular or myopathic muscle disease and is usually associated with chronic multisystem sarcoidosis [57]. The available cohorts are small and often monocentric, precluding any reliable sex-stratified risk estimation for these manifestations [56,57].

Finally, other rare localizations—such as central nervous system and renal sarcoidosis—are only sporadically reported in the available literature and were not specifically analysed in a sex-stratified manner in the cohorts included in this review, due to their very low prevalence and limited sample size.

#### 3.1.3. Sex Differences in Pulmonary Sarcoidosis

Sex-based differences in sarcoidosis are evident not only in the frequency and distribution of organ involvement but also in the clinical manifestations of pulmonary disease.

Some studies have reported sex-related differences in disease severity as assessed by Scadding stages, with women more frequently diagnosed at stages I–II, and men showing a higher prevalence of parenchymal involvement and fibrosis (stages III–IV) [34,55,58]. In the large Swedish cohort of 1429 patients, men presented significantly more often with radiographic stage II disease at diagnosis (46% vs. 36%), whereas women more frequently exhibited stage 0–I disease, confirming a trend toward more advanced pulmonary involvement at presentation in males [34]. However, findings are not entirely consistent across cohorts, and differences are not always statistically significant [37].

Respiratory function in sarcoidosis also appears to be influenced by biological sex. This concept has been investigated in a U.S. tertiary-care cohort of 602 patients, in which a restrictive impairment was the most frequent pulmonary function phenotype overall. Sex-stratified analyses demonstrated that men more frequently present with an obstructive spirometric pattern, whereas women tend to show a restrictive pattern [59]. These findings highlight that pulmonary functional phenotypes in sarcoidosis are heterogeneous and strongly modulated by both sex and race.

In addition, a more pronounced reduction in DLCO has been observed in female patients, particularly among smokers, suggesting a combined effect of sex and environmental exposure on pulmonary diffusion capacity impairment in sarcoidosis [60]. This interaction was clearly demonstrated in a cohort of 518 sarcoidosis patients, in which cigarette smoking was associated with a significant reduction in DLCO, with the most marked impairment observed in female smokers, who also exhibited greater airflow limitation and more severe gas exchange abnormalities compared with non-smoking women [60]. These data support a synergistic detrimental effect of female sex and smoking on pulmonary functional impairment in sarcoidosis.

### 3.2. Gender Differences in Illness Perception and Quality of Life

Assessment of quality of life (QoL) in patients with respiratory diseases has gained increasing attention in recent years. In sarcoidosis, numerous studies have explored how gender-related factors may influence patients’ perception of the disease, symptom burden, and psychological well-being. Recent research highlights significant gender-related differences in quality of life among sarcoidosis patients, with women more frequently reporting greater physical symptom burden and psychological distress than men.

These findings have been further corroborated by a recent prospective single-centre Turkish study by Bardakci et al. including 189 patients with pulmonary sarcoidosis, in which female sex was significantly associated with lower SF-36 scores—particularly in the domains of pain, general health, vitality and mental health—and with higher fatigue severity compared with male patients [61].

A large study by Hinz et al., involving 1197 German patients with sarcoidosis, found that women were more prone to develop anxiety and depression. Psychological distress was also more common in patients reporting dyspnoea, multiple organ involvement, and comorbid chronic diseases. Younger age and lower educational level were additional factors associated with worse emotional outcomes. However, the study was limited by its reliance on self-reported questionnaire data and the absence of clinical parameters such as lung function, which prevented correlation between psychological status and disease severity [62]. Consistent with these findings, a study by Dudvarski-Ilić et al., which enrolled 202 patients (154 women and 48 men) without relevant comorbidities, assessed QoL differences before and after therapy. Prior to treatment, despite being predominantly in Scadding stage I or II, female patients reported significantly lower QoL scores, indicating that subjective disease burden may be more pronounced in women even at earlier stages. Following three months of therapy, women showed a significant improvement in physical domain scores whereas no meaningful changes were observed in emotional well-being or daily activity scores, suggesting that current treatments may have a limited effect on psychosocial dimensions in female patients [63].

Other studies have emphasized the impact of organic symptoms—rather than psychological factors—on QoL. A study by De Vries et al., conducted among members of the Dutch Sarcoidosis Society, emphasized that women reported a greater number of physical symptoms and a higher use of symptomatic medications—including analgesics, non-steroidal anti-inflammatory drugs, eye drops, and psychiatric/neurological drugs. Notably, corticosteroid use in female patients was associated with lower scores in domains such as energy, mobility, body image, and work capacity [64].

A prospective study by Gwadera et al. provides further evidence of gender-related differences in health-related QoL (HRQL) among sarcoidosis patients. Despite comparable disease activity and pulmonary function (including DLCO, FEV1, FVC, and FEV1/FVC ratio), women reported significantly lower overall HRQL—particularly in the domain of daily functioning. Additionally, they more frequently experienced symptoms such as headaches, skin and hair problems, weight gain, negative body image, and bodily pain, suggesting that these factors may disproportionately affect women’s perception of their disease [65].

A prospective cross sectional study by Bourbonnais et al., involving 221 patients, examined the relationship between QoL and functional parameters. Poorer performance at the six-minute walk test (6MWT) and reduced DLCO were significantly associated with lower QoL in both sexes. Notably, however, dyspnoea or air hunger was reported significantly more often by women, which may reflect gender-related aspects of symptom perception and reporting [66].

Multivariate analyses revealed that while DLCO and 6MWT distance predicted poor QoL in both sexes, subjective dyspnea represented an independent and stronger determinant of QoL impairment exclusively in women. Consistently, data from a large UK national sarcoidosis registry showed that women were significantly more likely to report fatigue and breathlessness than men, despite similar pulmonary function and radiological staging, and that breathlessness emerged as a major driver of immunosuppressive treatment initiation irrespective of objective lung impairment [67].

Taken together, these findings indicate that female patients with sarcoidosis tend to report a worse QoL compared to males, due to a combination of gender-related factors such as psychological distress, physical symptom burden, and possibly more severe or chronic disease forms.

**Table 1:** Observational studies investigating sex-related differences in clinical presentation, organ involvement, pulmonary function, extrapulmonary manifestations, cardiac involvement, and quality of life in sarcoidosis.

Author, Year	Country	Study Design	N	Population	Main Study Domain	Outcomes Assessed	Key Findings
Lundkvist et al., 2022 [34]	Sweden	Retrospective observational, multicentric	1429	Adults with pulmonary sarcoidosis	Biological sex and disease presentation	Age at diagnosis, radiological stage, extrapulmonary manifestations	Male sex was associated with younger age at diagnosis and more frequent radiological stage II, whereas female sex was associated with skin and salivary gland involvement.
Lill et al., 2016 [35]	Estonia	Retrospective observational, monocentric	230	Sarcoidosis outpatients	Biological sex and disease presentation	FEV1, DLCO, FVC, extrapulmonary involvement	Female sex was associated with older age, lower smoking prevalence, greater extrapulmonary and musculoskeletal involvement, lower FEV1 and DLCO, and higher FVC % predicted.
Ungprasert et al., 2017 [36]	USA	Retrospective observational, monocentric	345	Incident sarcoidosis cases	Biological sex and disease presentation	Age, pulmonary and extrapulmonary involvement, ACE, calcium	Female sex was associated with older age at diagnosis and higher frequency of uveitis and cutaneous involvement, whereas male sex was associated with more frequent respiratory symptoms.
Haraldsdóttir et al., 2021 [37]	Iceland	Retrospective observational, monocentric	418	Tissue verified sarcoidosis	Biological sex and disease presentation	Incidence, age, smoking	Female sex was associated with older age at diagnosis, whereas incidence rates were similar between sexes.
Varron et al., 2012 [38]	France	Retrospective observational, monocentric	100	Late onset and younger sarcoidosis	Biological sex and disease presentation	Clinical phenotype, survival, treatment	Female sex was associated with higher frequency of late onset sarcoidosis, with more frequent asthenia, uveitis, and skin lesions in older patients.
Dumas et al., 2016 [39]	USA	Prospective observational, multicentric	377	Female nurses with sarcoidosis	Biological sex and disease presentation	Prevalence and incidence	Female sex showed increasing sarcoidosis incidence with age, with markedly higher prevalence and incidence among Black women.
Yanardag et al., 2003 [40]	Turkey	Retrospective observational, monocentric	170	Cutaneous sarcoidosis	Biological sex and disease presentation	Skin phenotypes and lung involvement	Female sex showed marked predominance in cutaneous sarcoidosis, with erythema nodosum as the most frequent lesion.
Liu et al., 2017 [41]	Taiwan	Retrospective observational, monocentric	38	Biopsy proven cutaneous sarcoidosis	Biological sex and disease presentation	Demographics, comorbidities	Female sex was strongly predominant and associated with older age and facial involvement.
Grunewald & Eklund, 2007 [42]	Sweden	Prospective observational, monocentric	150	Löfgren syndrome	Biological sex and disease presentation	EN, ankle arthritis, outcome	Female sex was associated with higher frequency of erythema nodosum, whereas male sex was associated with isolated ankle arthritis.
Soheilian et al., 2004 [43]	Iran	Prospective observational, monocentric	544	Uveitis patients	Biological sex and disease presentation	Etiology and anatomy	Female sex was associated with a higher frequency of sarcoidosis among patients with intermediate uveitis.
Kitamei et al., 2009 [44]	Japan	Retrospective observational, monocentric	1240	Intraocular inflammation	Biological sex and disease presentation	Etiology, age, sex	Female sex predominated among sarcoidosis related uveitis, whereas male sex was associated with younger onset.
Chung et al., 2007 [45]	Taiwan	Retrospective observational, monocentric	60	Sarcoidosis with uveitis	Biological sex and disease presentation	CT, uveitis pattern	Female sex showed marked predominance with peak onset in the sixth decade and predominant posterior segment involvement.
Ohara et al., 1992 [46]	Japan	Retrospective observational, monocentric	159	Systemic sarcoidosis	Biological sex and disease presentation	Type of intraocular lesions	Ocular involvement was highly prevalent in both sexes, with iritis as the most frequent manifestation.
Lobo et al., 2003 [47]	UK	Retrospective observational, monocentric	75	Sarcoid uveitis	Biological sex and disease presentation	Visual outcome	Severe visual loss was more frequent in panuveitis and multifocal choroiditis than in anterior uveitis.
Williamson et al., 2025 [48]	USA	Retrospective observational, monocentric	455	Cardiac sarcoidosis	Biological sex and disease presentation	Symptoms, left ventricular ejection fraction (LVEF), arrhythmias, hospitalizations, survival	Female sex was associated with more severe symptoms at presentation, whereas survival and arrhythmic outcomes were similar between sexes.
Martusewicz Boros et al., 2016 [49]	Poland	Retrospective observational, monocentric	1375	Biopsy proven sarcoidosis	Biological sex and disease presentation	MRI confirmed cardiac involvement	Male sex was associated with a significantly higher prevalence of cardiac sarcoidosis.
Kalra et al., 2021 [50]	USA	Retrospective observational, monocentric	324	Suspected cardiac sarcoidosis	Biological sex and disease presentation	Late gadolinium enhancement (LGE), arrhythmias, death	Female sex was associated with fewer ventricular arrhythmias and less extensive LGE, while mortality did not differ between sexes.
Iso et al., 2023 [51]	Japan	Retrospective observational, multicentric	512	Cardiac sarcoidosis	Biological sex and disease presentation	Ventricular arrhythmias, LVEF, survival	Male sex was independently associated with a higher risk of potentially fatal ventricular arrhythmias.
Duvall et al., 2023 [52]	USA	Retrospective observational, monocentric	252	Cardiac sarcoidosis	Biological sex and disease presentation	Heart failure (HF), arrhythmias, LVAD, transplant, death	Female sex was associated with more frequent symptomatic heart failure, whereas male sex was independently associated with higher arrhythmic risk.
Ahmed et al., 2024 [53]	USA	Retrospective observational, multicentric	760	Cardiac sarcoidosis with ICD	Biological sex and disease presentation	Major adverse cardiovascular events (MACE), Acute kidney injury (AKI), Sudden cardiac death (SCD)	Male sex was associated with higher ICD utilization and a higher risk of sudden cardiac death, whereas female sex was independently associated with a lower adjusted risk of major adverse cardiovascular events and acute kidney injury.
Nakasuka et al., 2022 [54]	Japan	Retrospective observational, multicentric	430	Cardiac sarcoidosis with CRT	Biological sex and disease presentation	Mortality, HF death, arrhythmias, BNP, LVEF	Female sex was independently associated with better ventricular arrhythmia free and cardiac adverse event free survival after CRT, while heart failure death free survival was similar between sexes.
Judson et al., 2012 [55]	USA	Retrospective observational, monocentric	1774	Tertiary sarcoidosis clinic patients	Biological sex and disease presentation	Organ distribution, treatment	Male sex was associated with more lung and cardiac involvement, whereas female sex was associated with skin, ocular, hepatic, and lymph node involvement.
Brito Zerón et al., 2016 [56]	Spain	Retrospective observational, monocentric	175	Consecutive sarcoidosis	Biological sex and disease presentation	2014 WASOG organ involvement	Female sex was associated with a higher frequency of cutaneous and musculoskeletal involvement and a lower frequency of hypercalcaemia, whereas male sex was associated with higher pulmonary involvement.
Salari et al., 2014 [57]	Iran	Cross sectional, monocentric	30	Musculoskeletal sarcoidosis	Biological sex and disease presentation	Joint, bone, muscle involvement	Female sex predominated markedly and was associated with a high frequency of sarcoidal arthropathy.
Westney et al., 2007 [58]	USA	Cross sectional, monocentric	165	African American sarcoidosis	Biological sex and disease presentation	Scadding stage, comorbid illness	Female sex was associated with a higher burden of chronic comorbid illnesses, whereas male sex showed a trend toward more severe chest radiographic stages.
Sharp et al., 2023 [59]	USA	Retrospective observational, monocentric	602	Sarcoidosis with PFTs	Biological sex and disease presentation	FEV1, FVC, DLCO	Male sex was associated with a higher prevalence of obstructive lung disease, whereas female sex was associated with a higher prevalence of restrictive impairment.
Krell et al., 2012 [60]	USA	Prospective observational, monocentric	518	Biopsy proven sarcoidosis	Biological sex and disease presentation	DLCO, airflow limitation	Female sex and smoking were independently associated with a greater reduction in DLCO, with the most pronounced impairment observed in female smokers.
Bardakci et al., 2024 [61]	Turkey	Prospective observational, monocentric	189	Pulmonary sarcoidosis	Gender differences in illness perception and quality of life	SF 36, FAS, FSS, spirometry, ACE	Female sex was associated with significantly lower SF 36 scores and higher fatigue severity.
Hinz et al., 2012 [62]	Germany	Cross sectional, multicentric	1197	Sarcoidosis society members	Gender differences in illness perception and quality of life	HADS, dyspnoea, comorbidities	Female sex was associated with higher depression scores in univariate analysis.
Dudvarski Ilić et al., 2009 [63]	Serbia	Prospective observational, monocentric	202	Biopsy proven sarcoidosis	Gender differences in illness perception and quality of life	SHQ	Women had significantly lower emotional, physical and total SHQ scores before therapy and lower physical scores after treatment.
De Vries et al., 1999 [64]	The Netherlands	Cross sectional, multicentric	1026	Sarcoidosis society members	Gender differences in illness perception and quality of life	WHOQoL 100, symptom checklist, medication use	Women reported a higher symptom burden, lower physical and psychological quality of life, and higher use of analgesics, NSAIDs and ophthalmic treatments, whereas men more frequently used oral corticosteroids.
Gwadera et al., 2021 [65]	Poland	Prospective observational, monocentric	75	Non smoking sarcoidosis	Gender differences in illness perception and quality of life	SHQ	Women showed significantly lower total and physical SHQ scores despite no sex differences in lung function or activity.
Bourbonnais et al., 2010 [66]	USA	Cross sectional, monocentric	221	Biopsy proven sarcoidosis	Gender differences in illness perception and quality of life	SF 36, SHQ, PFT, 6MWT, Borg	Women had significantly lower HRQoL across all domains; predictors differed by sex.
Bączek et al., 2025 [67]	UK	Retrospective observational, multicentric	1071	Pulmonary sarcoidosis	Gender differences in illness perception and quality of life	Age, symptoms, comorbidities, lung function, treatment	Women were older at diagnosis and reported more fatigue and higher ESR, whereas men showed higher rates of lymphopenia, elevated ACE, arrhythmia and methotrexate use; male sex and non white ethnicity were independently associated with initiation of immunosuppressive treatment.

Legend: 6MWT: 6-min walking test, ACE: angiotensin-converting enzyme, AKI: acute kidney injury, BNP: B-type natriuretic peptide, CRT: cardiac resynchronization therapy, DLCO: diffusion lung capacity for carbon monoxide; ESR: erythrocyte sedimentation rate, FAS: fatigue assessment scale, FEV1: forced expiratory volume in 1 s, FSS: fatigue severity scale, FVC: forced vital capacity, HADS: Hospital Anxiety and Depression Scale, HF: heart failure, HRQoL: health-related quality of life, ICD: implantable cardioverter defibrillator, LGE: late gadolinium enhancement, LVAD: left ventricular assist device, LVEF: left ventricular ejection fraction, MACE: major adverse cardiac events, MRI: magnetic resonance imaging, NSAID: non-steroidal anti-inflammatory drugs, PFT: pulmonary function test, SCD: sudden cardiac death, SF-36: short form 36 items, SHQ: Sarcoidosis Health Questionnaire, WASOG: World Association for Sarcoidosis and Other Granulomatous Disorder, WHOQoL: World Health Organization Quality of Life questionnaire.

## 4. Discussion

Sarcoidosis most commonly affects individuals between 20 and 50 years of age, with two distinct age-related incidence peaks: one in men aged 30–50 and another in women aged 50–60. This epidemiological discrepancy suggests a potential role of sex-specific factors in disease onset, as supported by observed differences in prevalence, disease severity, organ involvement, respiratory function, and radiological features [6,34].

Sex-based clinical differences have prompted investigation into the potential role of sex hormones in modulating disease expression and immune mechanisms. Epidemiological and pathogenic studies suggest a possible protective effect of estrogen on sarcoidosis, which may partly explain why women are more frequently diagnosed after menopause, when estrogen levels decrease markedly [22,68]. On this regard, it has been suggested that estrogenic exposure may delay the onset of sarcoidosis in women by influencing T-helper (Th)1/Th2 balance in immune response [69,70].

Indeed, non-caseating granuloma formation—the pathologic hallmark of sarcoidosis—is characterized by dominant expression of Th1 cytokines—interferon-γ, interleukin (IL)-2 and tumor necrosis factor-α—eliciting phagocyte-dependent inflammation, while Th2 cytokines (IL-4, IL-10) production is significantly impaired [70,71]. Accordingly, high-estrogen states, such as pregnancy, have been associated with a Th2-skewed immune response and reduced disease activity, whereas low-estrogen or androgenic states may favor a Th1-dominant profile, potentially facilitating disease onset or progression [68,72,73]. However, direct evidence linking circulating sex hormone levels to clinical outcomes in sarcoidosis remains limited.

Beyond immunological mechanisms, reproductive and hormonal life-history factors offer further insight into the potential protective role of estrogens [73]. Reproductive milestones and exogenous hormonal exposures may influence sarcoidosis risk; however, findings are inconsistent across populations, highlighting potential ethnic and geographic variability in hormone-disease interactions.

Further complicating the potential correlation between sarcoidosis onset and estrogenic status, exogenous estrogen administration, especially menopausal hormone therapy (MHT), has been associated with a higher risk of sarcoidosis [73], as well as other immune-related illnesses, like systemic lupus erythematosus and ulcerative colitis [74,75,76,77,78,79].

Regarding male patients with sarcoidosis, a reduced median circulating testosterone concentration was described when compared to healthy middle-aged men. In contrast, follicle-stimulating hormone (FSH) and luteinizing hormone (LH) levels appear unchanged, regardless of ethnicity or oral corticosteroid use. Persistently lower testosterone levels may therefore represent an additional biological factor contributing to sex-specific disease expression [80].

Recent genetic studies suggest that biological sex may influence susceptibility through distinct genetic pathways. A genome-wide admixture analysis in African Americans identified sex-specific associations in Löfgren’s syndrome: HLA-DQA1 in males and C6orf10 in females, along with non- HLA loci such as LRRTM4 and LNPEP, involved in immune and neuroendocrine regulation [81].

Some genetic signals were shared across sexes but showed different effect sizes, suggesting a modulatory role of biological sex rather than entirely distinct genetic mechanisms. These findings were validated in the UK Biobank and further modulated by ancestry. Notably, Löfgren’s syndrome showed broader genetic associations in females across the extended MHC region [81]. Although these genetic differences may contribute to clinical heterogeneity, their direct clinical translation remains limited.

Genetic differences in the sex groups could be linked to the observed differences in clinical manifestations in sarcoidosis male and female patients, as documented in various works. Overall, these results highlight the importance of considering both sex and ancestry in genetic studies, particularly given the central role of HLA genes in immune responses and the known female predominance in autoimmune conditions [81].

Beyond biological sex differences, gender-related factors in clinical presentation and health-related quality of life (HRQoL) are evident. Women more frequently report worse physical and psychological outcomes, including anxiety and depression, whereas men show lower emotional dysfunction and better adaptation to physical limitations [82,83]. Differences in pain perception, potentially influenced by sex hormones and genetic factors, may also contribute to these gender-related differences in disease experience [84,85].

It has been observed that women tend to report a higher level of pain sensitivity and a lower tolerance for discomfort. They also appear to be more inclined to seek assistance. Hormonal influences appear relevant, as testosterone has been described as protective, while estrogens and progesterone exert complex, cycle-dependent effects on pain perception [86,87]. Neuroimaging studies further suggest that hormonal modulation of pain-related brain networks, together with sex-specific genetic variants (e.g., MC1R), may partly explain the greater symptom burden reported by women with sarcoidosis [88]. It is possible that these factors, when taken together, may help to explain why women with sarcoidosis often report worse physical and psychological outcomes, reflecting gender-related perception of disease.

This review highlights that biological sex and gender-related factors are major contributors to the phenotypic heterogeneity of sarcoidosis, with clear implications for personalised medicine. Women more frequently exhibit extrapulmonary involvement and report greater symptom burden and reduced quality of life, which are influenced by gender-related factors such as psychological distress and social functioning. Men are more prone to parenchymal lung disease, fibrotic stages, and severe cardiac disease, with a higher risk of ventricular arrhythmias, which may more frequently necessitate ICD implantation. Recognizing these patterns allows clinicians to tailor monitoring, risk assessment, and therapeutic approaches according to the patient’s biological sex, gender-relates factors, and clinical phenotype, supporting individualized care strategies consistent with precision medicine.

Critical evaluation of the evidence is warranted. The included studies are heterogeneous in terms of study design, population characteristics, diagnostic criteria, and outcome assessment. Most studies were retrospective, limiting causal inference. Geographic and ethnic biases may affect generalizability, as several findings were predominantly based on specific populations (e.g., US Black women or Swedish cohorts). Moreover, some mechanistic interpretations, particularly regarding hormonal or genetic pathways, are speculative and not directly confirmed by the clinical data included in this review. Future research should prioritize prospective, multi-ethnic cohorts with standardized diagnostic and outcome measures to validate mechanistic hypotheses and generate robust, sex- and gender-specific evidence.

Building on these observations, the findings have important implications for clinical practice. Considering biological sex and gender-related factors in risk assessment, organ involvement monitoring, and treatment selection may help tailor clinical management to individual patient profiles. Tailored approaches, including potential hormone-based interventions, could optimize outcomes and reduce disease burden.

In conclusion, recognizing the differential disease expression between biological sexes and gender-related factors provides a foundation for developing more personalised approaches to sarcoidosis management. Integrating sex- and gender-specific considerations into monitoring, prevention, and treatment strategies can enhance care, particularly when combined with precision medicine approaches. Future research should continue refining these individualized strategies, ensuring interventions account for both biological and socio-cultural patient characteristics.

## Data Availability

No new data were created or analyzed in this study. Data sharing is not applicable to this article.

## Supplementary Materials

The following supporting information can be downloaded at: https://www.mdpi.com/article/10.3390/jpm16010024/s1, File S1: The PRISMA 2020 checklist; Table S1. Quality assessment of the included observational studies using the Newcastle–Ottawa Scale (NOS); Table S2. List of studies excluded after full-text screening, with year of publication and reason for exclusion.

## Abbreviations

The following abbreviations are used in this manuscript:

6MWT	6-min walking test
BMI	Body mass index
HRQL	Health-related quality of life
IL	interleukin
ICD	implantable cardioverter-defibrillator
ILD	Interstitial lung disease
LVEF	left ventricular ejection fraction
MHT	menopausal hormone therapy
QoL	Quality of life
RCT	Randomized clinical trial
