# Supplementary material for: Uncovering Sex and Gender Differences in Sarcoidosis: A Systematic Review of Current Evidence
Source: J Pers Med. 2026 Jan 5;16(1):24. doi: 10.3390/jpm16010024 (PMC12843133; doi:10.3390/jpm16010024)
Supplement: Supplementary file 1 [file jpm-16-00024-s001.zip › Supplementary material Table S1 and Table S2.pdf]

Supplementary Table S1. Quality assessment of the included observational studies using the Newcastle–Ottawa Scale (NOS). Studies were evaluated across three domains: Selection (maximum 4 stars), Comparability (maximum 2 stars), and Outcome (maximum 3 stars), with a total score ranging from 0 to 9. Overall risk of bias was classified as low (7–9 points), moderate (5–6 points), or moderate–high/high (≤4 points).

Author, year	Selection (4)	Comparability (2)	Outcome (3)	Total (/9)	Risk of bias
Ohara et al. 1992 [46]	★★☆☆ (2)	★☆ (1)	★☆☆ (1)	4/9	Moderate–high
De Vries et al. 1999 [64]	★★☆☆ (2)	★★ (2)	★★☆ (2)	6/9	Moderate
Yanardağ et al. 2003 [40]	★★★☆ (3)	☆☆ (0)	★★☆ (2)	5/9	Moderate
Lobo et al. 2003 [47]	★★★☆ (3)	★★ (2)	★★★ (3)	8/9	Low
Soheilian et al. 2004 [43]	★★☆☆ (2)	★☆ (1)	★☆☆ (1)	4/9	Moderate–high
Grunewald et al. 2007 [42]	★★★☆ (3)	★★ (2)	★★☆ (2)	7/9	Low–moderate
Westney et al. 2007 [58]	★★★☆ (3)	★☆ (1)	★★☆ (2)	6/9	Moderate
Dudvarski-Ilić et al. 2009 [63]	★★★☆ (3)	☆☆ (0)	★☆☆ (1)	4/9	Moderate–high
Bourbonnais et al. 2010 [66]	★★★★ (4)	★★ (2)	★★☆ (2)	8/9	Low
Hinz et al. 2012 [62]	★★☆☆ (2)	☆☆ (0)	★★☆ (2)	4/9	Moderate–high
Krell et al. 2012 [60]	★★★☆ (3)	☆☆ (0)	★★☆ (2)	5/9	Moderate
Judson et al. 2012 [55]	★★★☆ (3)	★★ (2)	★☆☆ (1)	6/9	Moderate
Varron et al. 2012 [38]	★★★☆ (3)	★★ (2)	★★☆ (2)	7/9	Low–moderate
Salari et al. 2014 [57]	★★★☆ (3)	☆☆ (0)	★☆☆ (1)	4/9	Moderate–high
Brito-Zerón et al. 2016 [56]	★★★☆ (3)	★★ (2)	★★★ (3)	8/9	Low
Dumas et al. 2016 [39]	★★★★ (4)	★★ (2)	★★★ (3)	9/9	Low
Lill et al. 2016 [35]	★★★☆ (3)	★★ (2)	★☆☆ (1)	6/9	Moderate
Martusewicz-Boros et al. 2016 [49]	★★★☆ (3)	★☆ (1)	★★☆ (2)	6/9	Moderate
Ungprasert et al. 2017 [36]	★★★★ (4)	★☆ (1)	★★☆ (2)	7/9	Low–moderate
Liu et al. 2017 [41]	★★★★ (4)	★★ (2)	★★☆ (2)	8/9	Low
Haraldsdóttir et al. 2021 [37]	★★★☆ (3)	★☆ (1)	★★☆ (2)	6/9	Moderate
Kalra et al. 2021 [50]	★★★☆ (3)	★☆ (1)	★★★ (3)	7/9	Low–moderate
Gwadera et al. 2021 [65]	★★★☆ (3)	☆☆ (0)	★★☆ (2)	5/9	Moderate
Lundkvist et al. 2022 [34]	★★★☆ (3)	★☆ (1)	★★☆ (2)	6/9	Moderate
Iso et al. 2023 [51]	★★★★ (4)	★★ (2)	★★☆ (2)	8/9	Low
Sharp et al. 2023 [59]	★★★☆ (3)	★☆ (1)	★★☆ (2)	6/9	Moderate
Duvall et al. 2023 [52]	★★★☆ (3)	★☆ (1)	★★★ (3)	7/9	Low–moderate
Nakasuka et al. 2023 [54]	★★★★ (4)	★☆ (1)	★★★ (3)	8/9	Low
Ahmed et al. 2024 [53]	★★★★ (4)	★★ (2)	★★☆ (2)	8/9	Low
Bardakci et al. 2024 [61]	★★★★ (4)	★☆ (1)	★★☆ (2)	7/9	Low–moderate
Bączek et al. 2025 [67]	★★★★ (4)	★★ (2)	★★★ (3)	9/9	Low
Williamson et al. 2025 [48]	★★★★ (4)	★★ (2)	★★★ (3)	9/9	Low

Supplementary Table S2. List of studies excluded after full-text screening, with year of publication and reason for exclusion. Studies were excluded due to high risk of bias (NOS ≤ 3), publication before 1990, review or editorial nature, case-report design, or because they were considered out of scope with respect to the objectives of the present review.

First Author	Title	Year	Reason for Exclusion
Henke et al.	The Epidemiology of Sarcoidosis in Rochester, Minnesota	1986	Published before 1990
Iwai et al.	Racial difference in cardiac sarcoidosis incidence observed at autopsy	1994	High risk of bias (NOS ≤ 3)
Vahid et al.	Scrotal Swelling and Sarcoidosis	2006	Case report
Spruit et al.	Hypogonadism in male outpatients with sarcoidosis	2007	Out of scope
Allen et al.	Vaginal Involvement in a Patient With Sarcoidosis	2010	Case report
Xu et al.	Sarcoidosis: vaginal wall and vulvar involvement	2012	Case report
Cozier et al.	Reproductive and Hormonal Factors in Relation to Incidence of Sarcoidosis in US Black Women	2012	Out of scope
Alyasin et al.	Genital Sarcoidosis as the Presenting Feature of the Disease	2013	Case report
Birnbaum et al.	Sarcoidosis: Sex-Dependent Variations in Presentation and Management	2014	Review

Knisely et al.	Vaginal sarcoidosis without other organ involvement in a patient with a history of endometrial cancer	2018	Case report
Giovannini et al.	Common symptoms for a rare disease in a girl with sarcoidosis: a case report	2018	Case report
De Cinque et al.	Testicular Sarcoidosis: The Diagnostic Role of Contrast-Enhanced Ultrasound	2021	Case report
Sodhi et al.	Sex and Gender in Lung Diseases and Sleep Disorders: A State-of-the-Art Review: Part 2	2022	Review
Dehara et al.	Reproductive and hormonal risk factors for sarcoidosis: a nested case–control study	2022	Out of scope
Singha et al.	The influence of age and sex in sarcoidosis	2022	Review
Xiong et al.	Sex differences in the genetics of sarcoidosis across European and African ancestry populations	2023	Out of scope
Gupta et al.	Exploring Race and Sex Differences in Cardiac Sarcoidosis: "Dispare" no More	2023	Editorial
Hassan et al.	Scrotal Sarcoidosis: A Case Report With Radiological Insights	2024	Case report
Moor et al.	Sexual Dysfunction in Patients With Sarcoidosis	2024	Out of scope
Dehara et al.	Menopausal hormone therapy and risk of sarcoidosis: a population-based nested case–control study in Sweden	2024	Out of scope
Griffin.	Sex differences in cardiac sarcoidosis	2023	Editorial
Utsunomiya-Nishimizu et al.	Uterine Manifestation of Sarcoidosis Diagnosed in the Setting of Fever of Unknown Origin	2025	Case report
Assayag et al.	Racial, ethnic, sex and gender equity for inclusive interstitial lung disease research	2025	Out of scope

Reference

1. Henke, C.E.; Henke, G.; Elveback, L.R.; Beard, M.; Ballard, D.J.; Kurland, L.T. The Epidemiology of Sarcoidosis in Rochester, Minnesota: A Population-Based Study of Incidence and Survival1. *Am. J. Epidemiology* **1986**, *123*, 840–845, https://doi.org/10.1093/oxfordjournals.aje.a114313.
2. Iwai, K.; Sekiguti, M.; Hosoda, Y.; A DeRemee, R.; Tazelaar, H.D.; Sharma, O.P.; Maheshwari, A.; I Noguchi, T. Racial difference in cardiac sarcoidosis incidence observed at autopsy. **1994**, *11*, 26–31.
3. Vahid, B., Weibel, S., Nguyen, C. Scrotal Swelling and Sarcoidosis. *Am J Med* **2006**, *119*(11):e3. https://doi.org/10.1016/j.amjmed.2006.02.004. PMID: 17071149.
4. Spruit, M.A.; Thomeer, M.J.; Gosselink, R.; Wuyts, W.A.; Van Herck, E.; Bouillon, R.; Demedts, M.G.; Decramer, M. Hypogonadism in male outpatients with sarcoidosis. *Respir. Med.* **2007**, *101*, 2502–2510, https://doi.org/10.1016/j.rmed.2007.07.009.
5. Allen, S.L.; Judson, M.A. Vaginal Involvement in a Patient With Sarcoidosis. *Chest* **2010**, *137*, 455–456, https://doi.org/10.1378/chest.09-0076.
6. Xu, F.; Cheng, Y.; Diao, R.; Zhou, X.; Wang, X.; Ma, Y.; Lv, W.; Shen, H. Sarcoidosis: Vaginal wall and vulvar involvement.. **2012**, *29*, 151–154.
7. Cozier, Y.C.; Berman, J.S.; Palmer, J.R.; Boggs, D.A.; Wise, L.A.; Rosenberg, L. Reproductive and Hormonal Factors in Relation to Incidence of Sarcoidosis in US Black Women: The Black Women's Health Study. *Am. J. Epidemiology* **2012**, *176*, 635–641, https://doi.org/10.1093/aje/kws145.
8. Soheila Alyasin, Reza Amin,Reza Amin, Sepideh Darougar, Sepideh DarougarGenital Sarcoidosis as the Presenting Feature of the Disease, Vol. 14, issue 1,; 76-80,Published online:Mar 25, 2013, https://brieflands.com/journals/semj/articles/20345.
9. Birnbaum, A.D.; Rifkin, L.M. Sarcoidosis: Sex-Dependent Variations in Presentation and Management. *J. Ophthalmol.* **2014**, *2014*, 1–7, https://doi.org/10.1155/2014/236905.
10. Knisely, A.; Girton, M.; Hintz, H.; Modesitt, S. Vaginal sarcoidosis without other organ involvement in a patient with a history of endometrial cancer: A case report. *Gynecol. Oncol. Rep.* **2018**, *23*, 34–36, https://doi.org/10.1016/j.gore.2018.01.004.

11. Giovannini, M.; Luzzati, M.; Ferrara, G.; Buccoliero, A.M.; Simonini, G.; de Martino, M.; Cimaz, R.; Giani, T. Common symptoms for a rare disease in a girl with sarcoidosis: A case report. *Ital. J. Pediatr.* **2018**, *44*, 74, https://doi.org/10.1186/s13052-018-0517-6.
12. De Cinque, A.; Corcioni, B.; Rossi, M.S.; Franceschelli, A.; Colombo, F.; Golfieri, R.; Renzulli, M.; Gaudiano, C. Case Report: Testicular Sarcoidosis: The Diagnostic Role of Contrast-Enhanced Ultrasound and Review of the Literature. *Front. Med.* **2021**, *7*, https://doi.org/10.3389/fmed.2020.610384.
13. Sodhi, A.; Cox-Flaherty, K.; Greer, M.K.; Lat, T.I.; Gao, Y.; Polineni, D.; Pisani, M.A.; Bourjeily, G.; Glassberg, M.K.; D'ambrosio, C. Sex and Gender in Lung Diseases and Sleep Disorders. *Chest* **2022**, *163*, 366–382, https://doi.org/10.1016/j.chest.2022.08.2240.
14. Dehara, M.; Sachs, M.C.; Kullberg, S.; Grunewald, J.; Blomberg, A.; Arkema, E.V. Reproductive and hormonal risk factors for sarcoidosis: A nested case–control study. *BMC Pulm. Med.* **2022**, *22*, 1–9, https://doi.org/10.1186/s12890-022-01834-1.
15. Singha, A.; Kirkland, M.; Drake, W.; Crouser, E.D. The influence of age and sex in sarcoidosis. *Curr. Opin. Pulm. Med.* **2022**, *28*, 307–313, https://doi.org/10.1097/mcp.0000000000000882.
16. Xiong, Y.; Kullberg, S.; Garman, L.; Pezant, N.; Ellinghaus, D.; Vasila, V.; Eklund, A.; Rybicki, B.A.; Iannuzzi, M.C.; Schreiber, S.; et al. Sex differences in the genetics of sarcoidosis across European and African ancestry populations. *Front. Med.* **2023**, *10*, https://doi.org/10.3389/fmed.2023.1132799.
17. Gupta, R.; Bermudez, F.; Alexander, K.M.; Sheikh, F.H. Exploring Race and Sex Differences in Cardiac Sarcoidosis: "Dispare" no More. *J. Card. Fail.* **2023**, *29*, 1146–1149, https://doi.org/10.1016/j.cardfail.2023.06.006.
18. Hassan, Y.; O'DOherty, E.; Rewhorn, M.J.; Ho, D.H.; Gabr, A.H. Scrotal Sarcoidosis: A Case Report With Radiological Insights. *Cureus* **2024**, *16*, e71172, https://doi.org/10.7759/cureus.71172.
19. Moor, C.C.; Gur-Demirel, Y.; Koudstaal, T.; Miedema, J.R. Sexual Dysfunction in Patients With Sarcoidosis. *Chest* **2024**, *166*, 1473–1475, https://doi.org/10.1016/j.chest.2024.07.159.
20. Dehara, M.; Kullberg, S.; Bixo, M.; Sachs, M.C.; Grunewald, J.; Arkema, E.V. Menopausal hormone therapy and risk of sarcoidosis: A population-based nested case–control study in Sweden. *Eur. J. Epidemiology* **2024**, *39*, 313–322, https://doi.org/10.1007/s10654-023-01084-3.
21. Griffin, J.M. Sex differences in cardiac sarcoidosis. *Heart* **2023**, *109*, 1346–1347, https://doi.org/10.1136/heartjnl-2023-322610.
22. Utsunomiya-Nishimizu, R.; Tsutsui, Y.; Horinouchi, N.; Yoshimura, K.; Nishida, M.; Shiota, S.; Miyazaki, E. Uterine Manifestation of Sarcoidosis Diagnosed in the Setting of Fever of Unknown Origin. *Int. Med Case Rep. J.* **2025**, *ume 18*, 339–344, https://doi.org/10.2147/imcrj.s501279.
23. Assayag, D.; Adegunsoye, A.; Khor, Y.H.; Bonella, F.; Borie, R.; Buendia, I.; Chaudhuri, N.; Gibson, K.; Johannson, K.A.; Kaminski, N.; et al. Racial, Ethnic, Sex, and Gender Equity for Inclusive Interstitial Lung Disease Research: An Official American Thoracic Society Research Statement. *Am. J. Respir. Crit. Care Med.* **2025**, *211*, 2251–2267, https://doi.org/10.1164/rccm.202509-2336st.
